# Can *Sergentomyia* (Diptera, Psychodidae) play a role in the transmission of mammal-infecting *Leishmania*?

**DOI:** 10.1051/parasite/2016062

**Published:** 2016-12-06

**Authors:** Carla Maia, Jérôme Depaquit

**Affiliations:** 1 Global Health and Tropical Medicine, GHTM, Instituto de Higiene e Medicina Tropical, IHMT, Universidade de Nova Lisboa, UNL Rua da Junqueira, 100 Lisbon Portugal; 2 Université de Reims Champagne-Ardenne, ANSES, EA4688 – USC « Transmission Vectorielle et Épidemiosurveillance de Maladies Parasitaires (VECPAR) », SFR Cap Santé, Faculté de Pharmacie 51 rue Cognacq-Jay 51096 Reims France

**Keywords:** *Leishmania* spp., leishmaniases, *Sergentomyia* sp., vector role

## Abstract

Leishmaniases are parasitic diseases caused by protozoa of the genus *Leishmania*. The parasites, which infect various wild and domestic mammals, including humans, are transmitted by the bite of phlebotomine sand flies belonging to the *Phlebotomus* genus in the Old World and to several genera (including *Lutzomyia*, *Psychodopygus* and *Nyssomyia*) in the New World. In this paper, we consider the genus *Sergentomyia* as divided into seven subgenera, mainly based on spermathecal morphology: *Sergentomyia*, *Sintonius*, *Parrotomyia*, *Rondanomyia*, *Capensomyia*, *Vattieromyia* and *Trouilletomyia*. We also include the groups *Grassomyia* and *Demeillonius* but exclude the genera *Spelaeomyia* and *Parvidens*. The possible role of *Sergentomyia* in the circulation of mammalian leishmaniases in the Old World has been considered as *Leishmania* DNA and/or parasites have been identified in several species. However, several criteria must be fulfilled to incriminate an arthropod as a biological vector of leishmaniasis, namely: it must be attracted to and willing to feed on humans and any reservoir host, and be present in the same environment; several unambiguously identified wild female flies not containing blood meals have to be found infected (through isolation and/or typing of parasites) with the same strain of *Leishmania* as occurs in humans or any reservoir host; the presence of infective forms of *Leishmania* on naturally infected females and/or on colonized sand flies infected experimentally should be observed; and finally, the vector has to be able to transmit parasites as a result of blood-feeding on a susceptible mammal.

## Abstract (fr)

Les leishmanioses sont des maladies causées par des protozoaires du genre *Leishmania*. Ces parasites infestent divers mammifères sauvages et domestiques incluant l’Homme. Ils sont transmis par la piqûre de phlébotomes appartenant au genre *Phlebotomus* dans l’Ancien Monde et à divers genres (*Lutzomyia*, *Psychodopygus*, *Nyssomyia*) dans le Nouveau Monde. Dans le cadre de ce travail, nous considérons par convention que le genre *Sergentomyia* contient sept sous-genres principalement définis par la structure des spermathèques : *Sergentomyia*, *Sintonius*, *Parrotomyia*, *Rondanomyia*, *Capensomyia*, *Vattieromyia* et *Trouilletomyia*. Nous considérons également les *Grassomyia* et les *Demeillonius* mais nous excluons les genres *Spelaeomyia* et *Parvidens*. Le rôle possible de *Sergentomyia* dans la transmission de leishmanioses dans l’Ancien Monde est de plus en plus évoqué depuis que des promastigotes ont été observées et que de l’ADN leishmanien a été mis en évidence chez plusieurs espèces du genre. Cependant, plusieurs critères doivent être validés pour incriminer un invertébré comme vecteur de leishmaniose : il doit être attiré et capable de prendre ses repas sanguins sur l’Homme et sur les réservoirs, et être présent dans le même environnement ; plusieurs femelles non gorgées doivent avoir été trouvées infestées (par isolement puis typage des parasites) avec la même souche leishmanienne que celle isolées chez l’Homme et les réservoirs ; la présence de formes infestantes doit être observée chez des femelles sauvages ou infestées expérimentalement; enfin, le vecteur doit être capable de transmettre le parasite, par piqûre infestante, à un hôte susceptible.

## Introduction

Leishmaniases are parasitic diseases caused by protozoa belonging to the family Trypanosomatidae, genus *Leishmania* (Ross, 1903), infecting several mammal species, including humans. Human leishmaniases have diverse clinical manifestations. Visceral leishmaniasis (VL), caused by parasites of *Leishmania Donovani* complex (*L. donovani* (Laveran & Mesnil, 1903) in the Old World and *L. infantum* (Nicolle, 1908) in both the Old and New Worlds), is a severe disease of humans and other mammals which leads to death if left untreated. A number of different species of *Leishmania* cause cutaneous leishmaniasis (CL) or mucocutaneous leishmaniasis, which are responsible for considerable morbidity in a vast number of people in endemic foci. Leishmaniases are endemic in 98 countries on 4 continents, with more than 350 million people at risk. Published figures indicate an estimated incidence of 0.2–0.4 million VL cases and 0.7–1.3 million CL cases [3, 42].

Parasites are transmitted by the bite of an insect vector, the phlebotomine sand fly (order Diptera, family Psychodidae; subfamily Phlebotominae) of the genus *Phlebotomus* (Rondani & Berté, in Rondani 1840) in the Old World and of several genera (including *Lutzomyia* (França, 1924), *Psychodopygus* (Mangabeira, 1941) and *Nyssomyia* (Barretto, 1962)) in the New World [reviewed by 19, 26, 42].

Members of the genus *Sergentomyia* (França & Parrot 1920) [10] are widely distributed throughout the Old World, namely in Palearctic, Afrotropical, Oriental and Australasian regions, and in the Indian subregion. They are dominant species in tropical areas where *Phlebotomus* species are scarce or absent [reviewed by 2]. The species of this genus share the following characters: a mesanepisternum without setae, abdominal tergites 2–6 all or most usually carrying recumbent hairs, an usual 1/III–XV antennal formula in the males and 2/III–XV in the females, a cibarium with an armature of teeth and/or denticles more developed in females than in males, a single paramere, and a style with four terminal spines (or often 2 terminal and 2 subterminal) and an accessory spine [40]. However, there are some exceptions related to most of these characters and the genus *Sergentomyia* is in fact not clearly delimited. It seems difficult to define any strong synapomorphy supporting this group, which is in fact a catchall group.

## Materials and methods

The role of *Sergentomyia* in the circulation of pathogenic *Leishmania* to humans and animals is reviewed primarily based on a search of the scientific literature available in the PubMed database up to October 2016 by combining the following keywords: “*Sergentomyia* AND *Leishmania* OR Taxonomy”. Reference lists of the available articles were also searched for publications deemed as relevant to this review. In addition, the *Sergentomyia* genus was defined conventionally.

### Background on the systematics of the *Sergentomyia* genus

Due to the lack of a large-scale phylogenetic study on the systematics of the Old World sand fly, there is no synapomorphy of the *Sergentomyia* genus. Consequently, it is difficult to define this genus with precision; it is more a convention than a scientific group. The presence of cibarial teeth in the females of this genus is one of the most used characters to include species in the *Sergentomyia*. This is, however, not true. Some non-*Sergentomyia* species like *Ph. papatasi* (Scopoli, 1786), *Ph. argentipes* (Annandale & Brunetti in Annandale, 1908), *Ph. stantoni* (Newstead, 1914), *Ph. fertei* (Depaquit, Léger & Robert, 2002), *Ph. berentiensis* (Léger & Rodhain, 1978), and *Idiophlebotomus* (Quate & Fairchild, 1961) spp., exhibit many teeth or denticles, whereas *Se. anodontis* or *Se. bailyi campester* do not exhibit teeth or denticles evident to observe. Currently, the *Sergentomyia* genus can be divided into seven subgenera, mainly based on spermathecal morphology: *Sergentomyia* with smooth, thin-walled and wide spermathecae; *Sintonius* (Nitzulescu, 1931) and *Trouilletomyia* (Depaquit & Léger, 2014) with annealed spermathecae; *Parrotomyia* (Theodor, 1958) with elliptical capsule, smooth, thin- or thick-walled spermathecae; *Rondanomyia* (Theodor, 1958) with smooth and wide spermathecae; *Capensomyia* (Davidson, 1979) with convoluted spermathecae; and *Vattieromyia* (Depaquit, Léger & Robert, 2008). Many *Sergentomyia* species do not fall into these subgenera and remain ungrouped. Moreover, some authors include the taxa *Grassomyia* (Theodor, 1958) with spherical and spiny spermathecae and *Demeillonius* (Davidson, 1980) with irregularly transversely striated, apically provided with a projecting stalk spermathecae [25, 38] whereas others, like us, consider them to be genera [1, 4, 11]. Moreover, many species remain unclassified at the subgeneric level (the “ungrouped *Sergentomyia*”). To our knowledge, no authors [11, 25, 38] follow the position adopted by Artemiev [4] who proposed to create six subgenera: *Perfilievia*, *Pharynxomyia*, *Longicoxia*, *Brevidentia*, *Luzonomyia* and *Irianomyia*, including in these groups part of *Neophlebotomus* (França & Parrot, 1920), *Parrotomyia* and ungrouped species.

We consider the subgenus *Rondanomyia* as valid, and as not valid the subgenus *Neophlebotomus* according to the position adopted by Léger et al. [22]. Briefly, França & Parrot (1920) created the taxon *Neophlebotomus* to classify phlebotomine sand flies which are intermediate between *Phlebotomus* and *Sergentomyia*. Lewis [23] in a paper related to the phlebotomine sand flies of the oriental region, more probably misunderstanding than disagreeing with França & Parrot (1920), considered the subgenus *Neophlebotomus* as the senior synonym of *Rondanomyia*.

**Figure 1.**
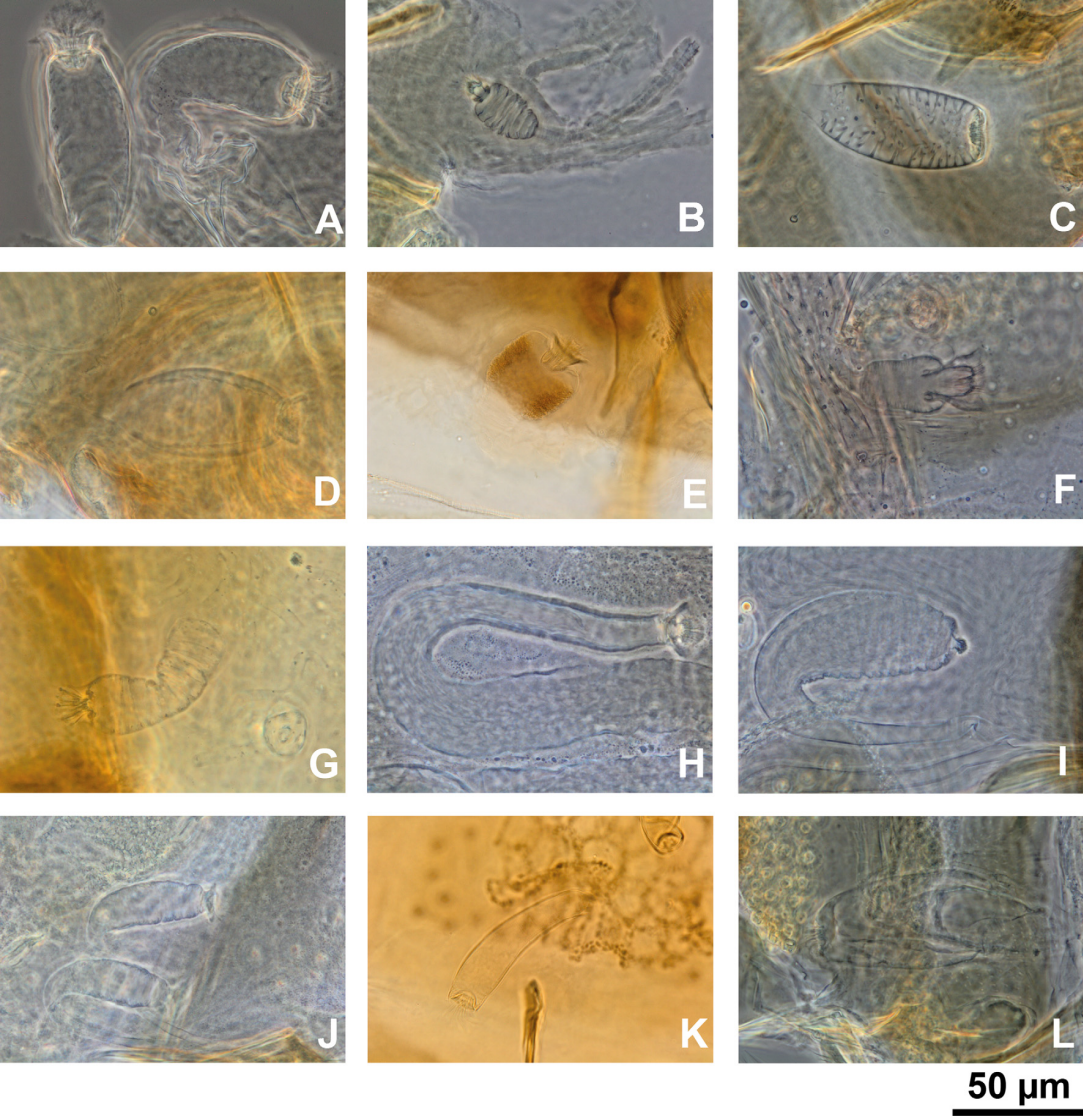
Spermathecae of *Sergentomyia* spp. s. l.: *Sergentomyia* (*Sergentomyia*) *minuta* (A); *Sergentomyia* (*Sintonius*) *clydei* (B); *Sergentomyia* (*Rondanomyia*) *goodmani* (C); *Sergentomyia* (*Parrotomyia*) *magna* (D); *Grassomyia* sp. from Madagascar (E); *Sergentomyia* (*Vattieromyia*) *anka* (F); *Demeillonius transvaalensis* (G), and the following ungrouped species: *Sergentomyia anodontis* (H); *Sergentomyia quatei* (I); *Sergentomyi asylvatica* (J); *Sergentomyia majungaensis* (K); and *Sergentomyia bailyi* (L).

**Figure 2.**
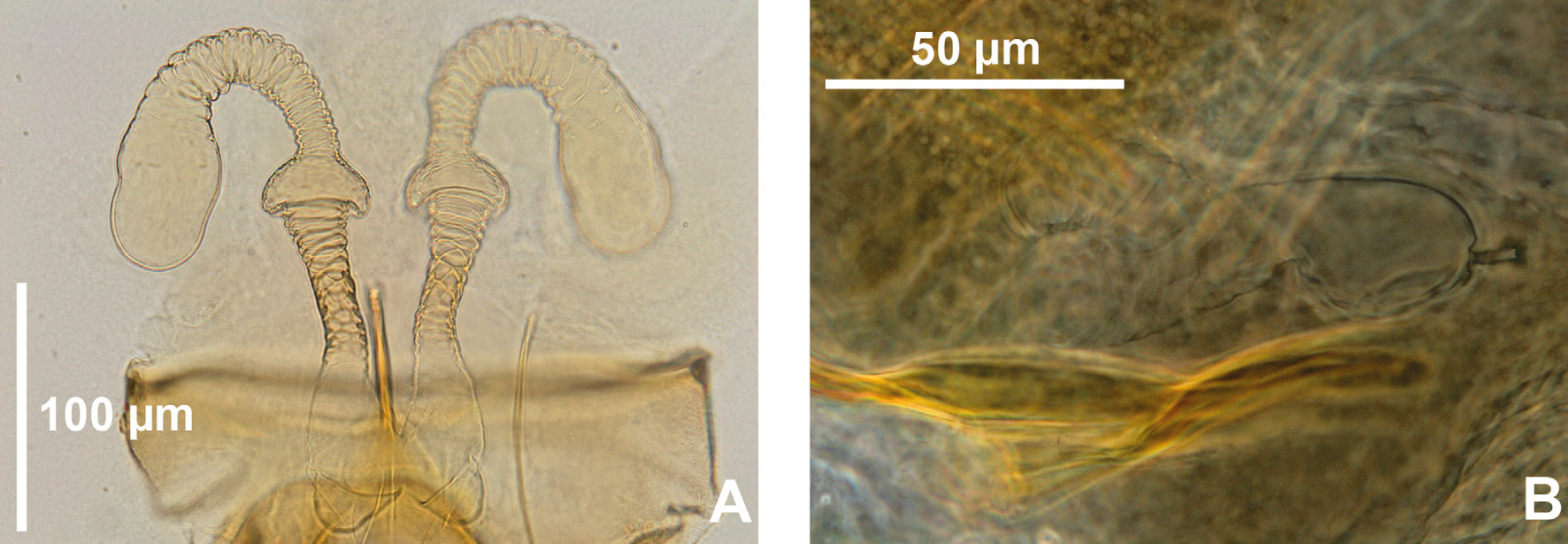
Spermathecae of *Spelaeomyia mirabilis* (A) and *Parvidens heischi* (B).

In fact, in this manuscript, pending a revision of this genus, we consider conventionally as *Sergentomyia sensu lato* the Old World species (Fig. 1) which do not belong to the genera *Phlebotomus*, *Idiophlebotomus*, *Australophlebotomus* (Theodor, 1948), *Spelaeophlebotomus* (Theodor, 1948), *Chinius* (Leng, 1987), *Spelaeomyia* (Theodor, 1948) and *Parvidens* (Theodor & Mesghali, 1964). The last ones (Fig. 2) cannot be considered as *Sergentomyia* s.l.

### *Sergentomyia* as possible vectors of *Leishmania* spp. pathogenic to humans and animals

As most *Sergentomyia* species feed preferentially on cold-blooded vertebrates, being proven vectors of reptile *Leishmania* species, it is generally accepted that they cannot transmit either *Leishmania* or any other pathogens to humans. However, based upon literature reviews, consideration of the role of *Sergentomyia* in the circulation of Old World *Leishmania* species with medical and veterinarian importance has been raised, but to fully prove the vector status of this Phlebotomine sand fly genus, several criteria need to be fulfilled [18], namely: (i) the vector must be found repeatedly infected in nature with the same *Leishmania* species as occurs in humans and any reservoir host(s), and this must be confirmed by comparison of isolates using isoenzymes and/or DNA; (ii) it must feed on humans and, in the case of zoonotic transmission, it must bite the reservoir host(s) as well; (iii) a strong ecological association between the vector, humans and any reservoir host should be evident; (iv) the vector must support the complete development of the parasite after the infecting blood meal has been digested, and (v) it must be able to transmit the parasite by bite to a susceptible host while taking a blood meal. In addition, and according to Ready [33], two more criteria based on mathematical modelling must be considered. The first one focused on demonstrating that the vector is essential for maintaining the transmission of the parasite, and the second on demonstrating a significant link between the decrease of disease prevalence and the decrease in biting density of the vector.

Indicative data about the potential role of some *Sergentomyia* species as a vector of mammalian *Leishmania* were given, namely the isolation of *Leishmania major* (Yakimoff & Shokkor, 1914) parasites from *Sergentomyia ingrami* (Newstead, 1914) collected in an endemic focus of cutaneous leishmaniasis in Kenya [28], and by the recent isolation of *L. infantum* from *Se. dubia* (Parrot, Mornet & Cadenat, 1945) and *Se. schwetzi* (Adler, Theodor & Parrot, 1929) in an endemic focus of canine leishmaniasis in Senegal [39]. *Leishmania* promastigotes have also been microscopically observed in several species of *Sergentomyia*: *Grassomyia affinis* (Theodor, 1933), *Grassomyia squamipleuris* (Newstead, 1912), *Se. africana* (Newstead, 1912), *Se. antennata* (Newstead, 1912), *Se. bedfordi* (Newstead, 1914), *Se. clydei* (Sinton, 1928), *Se. garnhami* (Heisch, Guggisberg & Teesdale, 1956), *Se. graingeri* (Heisch, Guggisberg & Teesdale, 1956), *Se. ingrami*, *Se. kirki* (Parrot, 1948), and *S. schwetzi*, in Kenya (reviewed by Kaddu et al. [15]) and in Ethiopia [13] but none of the parasites were biochemically or genetically typed and therefore they could not be confirmed to be *Leishmania* parasites.

Additional support for the potential role of *Sergentomyia* as a vector was provided by the detection of *Leishmania* DNA in several *Sergentomyia* species: that of *L. major* has been detected in *Se. clydei* and *Se. minuta* (Rondani, 1843) in Tunisia [5, 14], in *Se. minuta* in Portugal [7] and in *Se. sintoni* in Iran [31]. Despite the title of the publication, and the detection of *L. major* DNA in *Spelaeomyia darlingi* (Lewis & Kirk, 1954) in Mali [6], it is not possible to consider this species as a member of the genus *Sergentomyia*. In addition, *L. donovani* DNA has been detected in *Se. babu* (Annandale, 1910) in India [27], *L. infantum* DNA in *Se. dubia*, *Se. magna* (Sinton, 1932) and *Se. schwetzi* in Senegal [39] while “*Leishmania siamensis”* (*nomen nudum*) DNA has been found in *Se. barraudi* (Sinton, 1929) and in *Se. gemmea* (Lewis & Jeffery, 1978) in Thailand [17]. This so-called species, which belongs to the *Leishmania enrietti* (Muniz & Medina, 1948) complex, has not been formally named and described, and therefore is not taxonomically valid [2]. Moreover, it was recently shown by molecular tools that the majority of the strains previously described as “*L. siamensis*” may actually be *Leishmania martiniquensis* [32], the main causative agent of cutaneous leishmaniasis in Martinique Island (French West Indies) [8], including the *Leishmania* DNA sequenced from phlebotomine sand flies. Interestingly, it was recently experimentally proven that parasites of this complex developed late-stage infections in the biting midge *Culicoides sonorensis* (Wirth & Jones, 1957) [37] reinforcing the notion that vectors other than phlebotomine sand flies should be considered as part of epidemiological studies on *Leishmania* infecting mammals.

The detection of *Leishmania tropica* (Wright, 1903) DNA has been achieved from *Se. ingrami* and *Se. hamoni* (Abonnenc, 1958) collected in Ghana [30]. In addition, Maia et al. [24] also detected *Leishmania* sp. DNA phylogenetically related to those considered pathogenic to humans and dogs in *Se. minuta* collected in the South of Portugal.

Nevertheless, it is essential to keep in mind that PCR positivity alone should not be used for incrimination of a sand fly (or any other hematophagous arthropod) as a *Leishmania* vector, as the detection of DNA does not give any information about the parasites’ viability, nor about the presence as virulent metacyclic promastigotes, as the early phase of *Leishmania* development in the vector is non-specific and promastigotes are able to develop in various bloodsucking arthropods [reviewed by 36]. In fact, the detection of DNA of *Leishmania* through PCR-based tools has led to speculate about the vector competence of several “alternative” or “new” vectors [reviewed by 36]. For instance, the incrimination of biting midges as vectors of *L. enrietti* complex causing cutaneous leishmaniasis in red kangaroos [9] was the basis of the experimental infection of *Culicoides nubeculosus* with *L. infantum* and *L. major* parasites [35]; the authors demonstrated that, although both parasites species were able to develop early phases of infections, they were eliminated with the bloodmeal remnants; however, the DNA of both *Leishmania* species was detected until seven days post-infection, despite no living parasites being observed at that time point by microscopic examination, reinforcing the conclusion that the detection of *Leishmania* DNA does not prove the vector competence of any blood-sucking arthropod.

**Figure 3.**
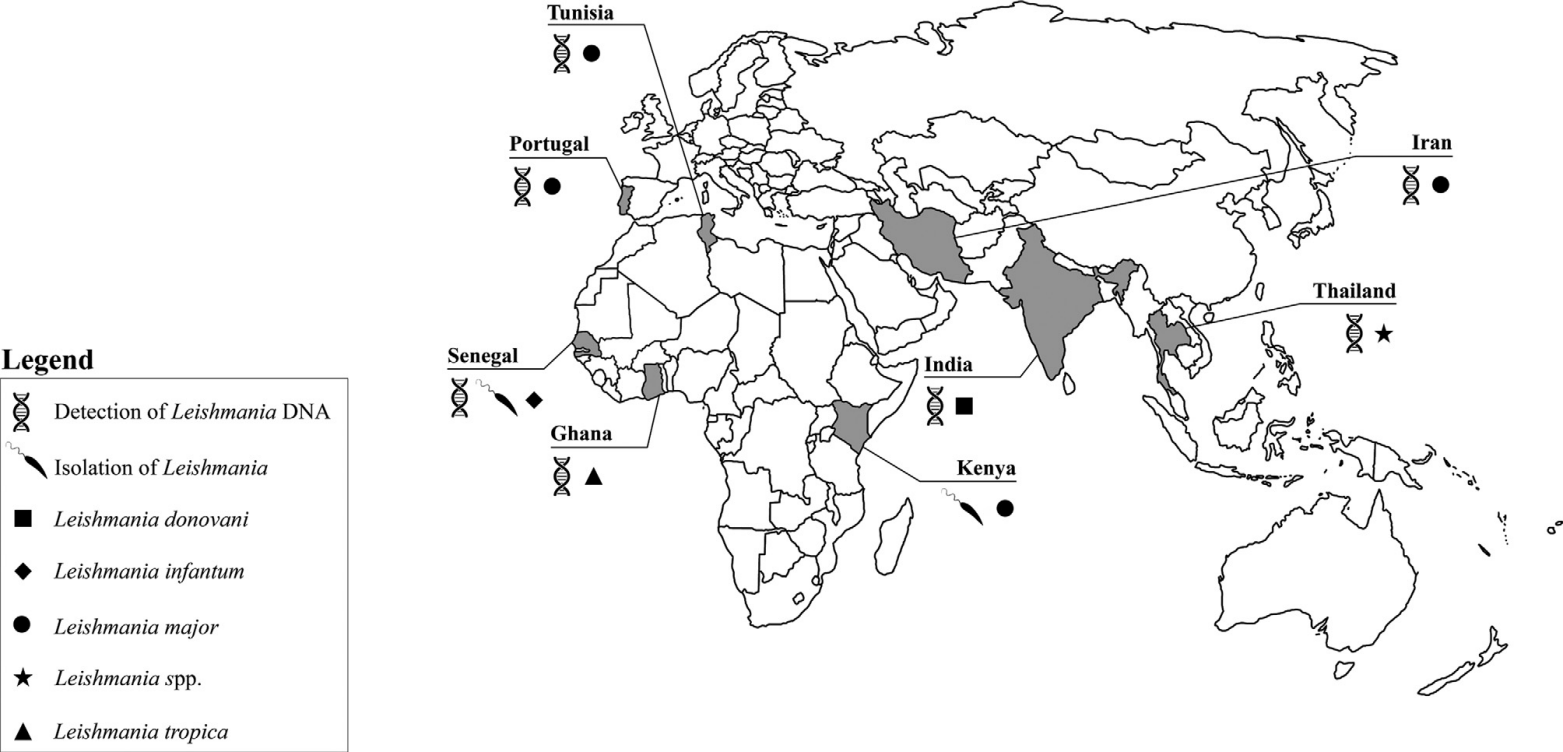
Detection of *Leishmania* parasites and/or DNA in *Sergentomyia* species in the Old World.

A primary determinant of vector competence in phlebotomine sand flies is the ability of *Leishmania* to survive defecation and to attach to midgut epithelium [reviewed by 16]. The direct microscopic observation of *Leishmania* promastigotes and their localization in the digestive tract is crucial before reaching any conclusion about the vectorial competence of an arthropod [reviewed by 36]. In fact, another criterion that a *Leishmania* vector should fulfil is the presence of parasites in the anterior midgut, on the stomodeal valve and the presence of infective forms on naturally infected females and/or on experimentally infected sand flies. The presence of flagellated parasites in dissected wild caught *Se. dubia* and *Se. schwetzi* females, which were subsequently successfully cultivated and characterized as *L. infantum*, was recently reported [39]. These results are contradictory to those obtained by Sadlova et al. [34] after testing the susceptibility of laboratory colonized Ethiopian *Se. schwetzi* to three *Leishmania* species capable of infecting humans (i.e. *L. donovani*, *L. infantum* and *L. major*). During early phases of infection, infection rates of all tested *Leishmania* species were very high (>90%) and comparable with those reached in control vectors (i.e. *Lutzomyia* (*Lutzomyia*) *longipalpis* (Lutz & Neiva, 1912) infected with *L. infantum* and *Phlebotomus* (*Phlebotomus*) *duboscqi* (Neveu-Lemaire, 1906) infected with *L. major*, respectively); however, none of them were able to develop successfully into late-stage infections. According to the authors, the refractoriness of this particular *Sergentomyia* species to the tested *Leishmania* species was probably related to the short period between the breakdown of the peritrophic matrix and the defecation of the bloodmeal remnants, thus avoiding the attachment of parasites to *Se. schwetzi* midgut epithelium. Similarly, in the study performed by Kaddu et al. [15], *Se. adleri*, *Se. ingrami* and *Se. schwetzi* did not support late-stage infections of *L. donovani*. Experimental data obtained by Kaddu et al. [15], Lawyer et al. [21] and Sadlova et al. [34] (i.e. the refractoriness of different *Sergentomyia* species to different *Leishmania* species pathogenic to humans) do not support conclusions based on field findings, namely the isolation of *L. major* from *Se. garnhami* [28] and *L. infantum* from *Se. dubia* and *Se. schwetzi* [39], confirming that the accidental results obtained in the field should always be interpreted with caution, and that more extensive studies focusing on isolation and typing of parasites from several unambiguously identified wild female flies not containing blood meals must be performed. In any case, the lack of vector competence of some African sand flies obtained under experimental conditions should not be extended to the whole of the genus as the competence and permissiveness of the different *Phlebotomus* species to different Old World *Leishmania* (e.g. *Phlebotomus papatasi* (Scopoli, 1786) is a proven vector of *L. major* but it is refractory to infection by *L. infantum*) has also been observed [reviewed by 16, 41]. All these findings have been summarized in Figure 3.

The demonstration that the sand fly is attracted to humans and displays biting behaviour towards humans and any reservoir host is also essential for it to be considered a vector. Despite most *Sergentomyia* species being herpetophilic, some of them found infected with *Leishmania* pathogenic to humans (e.g. *Sa. clydei*, *Sa. darlingi*, *Sa. minuta*, *Sa. schwetzi*) have been reported to feed on mammals, including man [5, 6, 13, 24, 29, 39].

Another criterion is a strong ecological association between the phlebotomine sand fly, which should be abundant in the endemic areas of the disease, and man as well as any reservoir host. This association has been reported for *Se. schwetzi*, which seems to be the predominant phlebotomine sand fly species caught in some endemic foci of VL in Sudan [20] and Ethiopia [12], together with strong endophilic [20] and human-biting behaviours [13]. Similar results were recently obtained by Senghor et al. [39] in a focus of canine leishmaniasis caused by *L. infantum* where the presence of *Se. dubia* and *Se. schwetzi* was strongly associated with humans and dogs, and where the probability of infection was higher indoors and in peridomestic environments for *Se. dubia* sand flies and in peridomestic areas for *Se. schwetzi*. In addition, there was a significant correlation between the detection of *L. infantum* DNA in both *Sergentomyia* species with the presence of antibodies against the parasite in dogs and/or humans. Interestingly, in many foci where the role of *Sergentomyia* spp. is evoked, the classical *Phlebotomus* vectors are absent or scarce (West Africa, South-East Asia), whereas they are abundant and sympatric in some foci (Iran, Tunisia, Kenya, Portugal).

A fifth criterion is that the suspected vector will become infected by biting and feeding on the reservoir host or an equivalent laboratory model (xenodiagnosis). In the attempt made by Lawyer et al. [21] with laboratory reared Kenyan *Se. schwetzi* that fed on a lesion on the nose of a hamster infected with *L. major*, parasites multiplied slowly in the phlebotomine sand fly midgut, but did not migrate anteriorly nor survive beyond 90 h post-feeding. More studies evaluating the infectiousness of *Leishmania-*infected vertebrate hosts to laboratory reared *Sergentomyia* species are needed. Nevertheless, a major limitation to experimentally evaluate whether *Sergentomyia* spp. fulfils this criterion is the worldwide lack of *Sergentomyia* colonies other than *Se. schwetzi*, stressing that more efforts are needed to set up laboratory colonies of *Sergentomyia* species that have been incriminated in the transmission of mammal-infecting *Leishmania*.

Despite the importance of demonstrating that a potential vector is experimentally capable of transmitting parasites as a result of blood-feeding on mammals, this criterion is difficult to assess because phlebotomine sand flies must first be infected, then need to survive after blood digestion and must feed again on a non-infected susceptible host, a procedure that is quite difficult to accomplish [reviewed by 26]. This is probably the reason why the only attempt to partially demonstrate this criterion was performed by Mutinga et al. [28], who inoculated *L. major* parasites isolated from *Se. garnhami* in BALB/c mice and observed the development of the typical cutaneous lesions with the presence of numerous amastigotes. Despite the laboriousness of the experiments, determining the ability of *Sergentomyia* spp. to transmit *Leishmania* to a naïve mammal host is of crucial importance.

## Conclusion

To sum up, some of the requirements needed for vectorial incrimination have been observed in *Sergentomyia* spp. such as: (i) epidemiological overlapping of the geographical distributions of *Se. dubia* and *Se. schwetzi* and canine/human *Leishmania* seroprevalence; (ii) evidence of anthropophilic behaviour (e.g. *Se. minuta*, *Se. schwetzi*); (iii) evidence that *Se. dubia*, *Se. schwetzi* and *Se. ingrami* support natural infections with promastigotes of the same *Leishmania* species as occurs in humans and reservoir hosts (i.e. *L. infantum* and *L. major*). Future work must be done to unravel whether and which *Sergentomyia* species accomplish all criteria needed to be incriminated as a vector of Old World *Leishmania* species pathogenic to mammals.

## References

[1] Abonnenc E, Léger N. 1976 Sur une classification rationnelle des Diptères Phlebotomidae. Cahiers de l’O.R.S.T.O.M. Série Entomologie Médicale et Parasitologie, 14, 69–78.

[2] Akhoundi M, Kuhls K, Cannet A, Votýpka J, Marty P, Delaunay P, Sereno D. 2016 A historical overview of the classification, evolution, and dispersion of *Leishmania* parasites and sandflies. PLoS Neglected Tropical Diseases, 10, e0004349. 26937644 10.1371/journal.pntd.0004349 PMC4777430

[3] Alvar J, Vélez I, Bern C, Herrero M, Desjeux P, Cano J, Jannin J, den Boer M, WHO Leishmaniasis Control Team. 2012 *Leishmaniasis* worldwide and global estimates of its incidence. PLoS One, 7, e35671. 22693548 10.1371/journal.pone.0035671 PMC3365071

[4] Artemiev M. 1991 A classification of the subfamily Phlebotominae. Parassitologia, 33, 69–77. 1841259

[5] Ayari C, Ben Othman S, Chemkhi J, Tabbabi A, Fisa R, Ben Salah A, BenAbderrazak S. 2016 First detection of *Leishmania major* DNA in *Sergentomyia* (*Sintonius*) *clydei* (Sinton, 1928, Psychodidae: Phlebotominae), from an outbreak area of cutaneous leishmaniasis in Tunisia. Infection, Genetics and Evolution, 39, 241–248. 10.1016/j.meegid.2015.10.030 26538476

[6] Berdjane-Brouk Z, Koné AK, Djimdé AA, Charrel RN, Ravel C, Delaunay P, del Giudice P, Diarra AZ, Doumbo S, Goita S, Thera MA, Depaquit J, Marty P, Doumbo OK, Izri A. 2012 First detection of *Leishmania major* DNA in *Sergentomyia (Spelaeomyia) darlingi* from cutaneous leishmaniasis foci in Mali. PLoS One, 7, e28266. 22276095 10.1371/journal.pone.0028266 PMC3262778

[7] Campino L, Cortes S, Dionísio L, Neto L, Afonso MO, Maia C. 2013 The first detection of *Leishmania major* in naturally infected *Sergentomyia minuta* in Portugal. Memórias do Instituto Oswaldo Cruz, 108, 516–518. 23828004 10.1590/0074-0276108042013020 PMC3970624

[8] Desbois N, Pratlong F, Quist D, Dedet JP. 2014 *Leishmania* (*Leishmania*) *martiniquensis* n. sp. (Kinetoplastida: Trypanosomatidae), description of the parasite responsible for cutaneous leishmaniasis in Martinique Island (French West Indies). Parasite, 21, 12. 24626346 10.1051/parasite/2014011 PMC3952653

[9] Dougall AM, Alexander B, Holt DC, Harris T, Sultan AH, Bates PA, Rose K, Walton SF. 2011 Evidence incriminating midges (Diptera: Ceratopogonidae) as potential vectors of *Leishmania* in Australia. International Journal for Parasitology, 41, 571–579. 21251914 10.1016/j.ijpara.2010.12.008

[10] França C, Parrot L. 1920 Introduction à l’étude systématique des Diptères du genre *Phlebotomus*. Bulletin de la Société de Pathologie Exotique, 13, 695–708.

[11] Galati E. 2016 Phlebotominae (Diptera, Psychodidae) Classificação, Morfologia, Terminologia e Identificação de Adultos. Apostila. Bioecologia e Identificação de Phlebotominae. Volume 1 Universidade de São Paulo: São Paulo, Brasil p. 131.

[12] Gebre-Michael T, Lane RP. 1996 The roles of *Phlebotomus martini* and *P. celiae* (Diptera: Phlebotominae) as vectors of visceral leishmaniasis in the Aba Roba focus, southern Ethiopia. Medical and Veterinary Entomology, 10, 53–62. 8834743 10.1111/j.1365-2915.1996.tb00082.x

[13] Hailu A, Balkew M, Berhe N, Meredith S, Gemetchu T. 1995 Is *Phlebotomus* (*Larroussius*) *orientalis* a vector of visceral leishmaniasis in south-west Ethiopia? Acta Tropica, 60, 15–20. 8546034 10.1016/0001-706x(95)00093-t

[14] Jaouadi K, Ghawar W, Salem S, Gharbi M, Bettaieb J, Yazidi R, Harrabi M, Hamarsheh O, Ben Salah A. 2015 First report of naturally infected *Sergentomyia minuta* with *Leishmania major* in Tunisia. Parasite & Vectors, 8, 649. 10.1186/s13071-015-1269-4 PMC4687309 26692017

[15] Kaddu JB, Mutinga MJ, Nyamori MP. 1986 *Leishmania* in Kenyan phlebotomine sandflies. 4. Artificial feeding and attempts to infect 6 species of laboratory-reared sandflies with *Leishmaniadonovani*. Insect Science and its Application, 7, 731–735.

[16] Kamhawi S. 2006 Phlebotomine sand flies and *Leishmania* parasites: friends or foes? Trends in Parasitology, 22, 439–445. 16843727 10.1016/j.pt.2006.06.012

[17] Kanjanopas K, Siripattanapipong S, Ninsaeng U, Hitakarun A, Jitkaew S, Kaewtaphaya P, Tan-ariya P, Mungthin M, Charoenwong C, Leelayoova S. 2013 *Sergentomyia* (*Neophlebotomus*) *gemmea*, a potential vector of *Leishmania siamensis* in southern Thailand. BMC Infectious Diseases, 13, 333. 23870062 10.1186/1471-2334-13-333 PMC3725172

[18] Killick-Kendrick R. 1990 Phlebotomine vectors of the leishmaniasis: a review. Medical and Veterinary Entomology, 4, 1–24. 2132963 10.1111/j.1365-2915.1990.tb00255.x

[19] Killick-Kendrick R. 1999 The biology and control of phlebotomine sand flies. Clinics in Dermatology, 17, 279–289. 10384867 10.1016/s0738-081x(99)00046-2

[20] Lambert M, Dereure J, El-Safi SH, Bucheton B, Dessein A, Boni M, Feugier E, Dedet JP. 2002 The sandfly fauna in the visceral-leishmaniasis focus of Gedaref, in the Atbara-River area of eastern Sudan. Annals of Tropical Medicine and Parasitology, 96, 631–636. 12396326 10.1179/000349802125001474

[21] Lawyer PG, Ngumbi PM, Anjili CO, Odongo SO, Mebrahtu YB, Githure JI, Koech DK, Roberts CR. 1990 Development of *Leishmania major* in *Phlebotomus duboscqi* and *Sergentomyia schwetzi* (Diptera: Psychodidae). American Journal of Tropical Medicine and Hygiene, 43, 31–43. 2382763 10.4269/ajtmh.1990.43.31

[22] Léger N, Depaquit J, Robert V. 2005 Les phlébotomes de Madagascar (Diptera: Psychodidae). IV. Description de *Sergentomyia* (*Rondanomyia*) *goodmani* n. sp. Rétablissement du sous-genre *Rondanomyia* Theodor. Parasite, 12(1), 51–57. 15828582 10.1051/parasite/2005121051

[23] Lewis DJ. 1978 The phlebotomine sandflies (Diptera: Psychodidae) of the Oriental Region. Bulletin of the British Museum (Natural History), Entomology Series, 37(6), 217–343.

[24] Maia C, Parreira R, Cristóvão JM, Freitas FB, Afonso MO, Campino L. 2015 Molecular detection of *Leishmania* DNA and identification of blood meals in wild caught phlebotomine sand flies (Diptera: Psychodidae) from southern Portugal. Parasites & Vectors, 8, 173. 25889732 10.1186/s13071-015-0787-4 PMC4377202

[25] Marcondes CB. 2007 A proposal of generic and subgeneric abbreviations for Phlebotomine sandflies (Diptera: Psychodidae: Phlebotominae) of the World. Entomological News, 118, 351–356.

[26] Maroli M, Feliciangeli MD, Bichaud L, Charrel RN, Gradoni L. 2013 Phlebotomine sandflies and the spreading of leishmaniases and other diseases of public health concern. Medical and Veterinary Entomology, 27, 123–147. 22924419 10.1111/j.1365-2915.2012.01034.x

[27] Mukherjee S, Hassan MQ, Ghosh A, Ghosh KN, Bhattacharya A, Adhya S. 1997 *Leishmania* DNA in *Phlebotomus* and *Sergentomyia* species during a kala-azar epidemic. American Journal of Tropical Medicine and Hygiene, 57, 423–425. 9347957 10.4269/ajtmh.1997.57.423

[28] Mutinga MJ, Massamba NN, Basimike M, Kamau CC, Amimo FA, Onyido AE, Omogo DM, Kyai FM, Wachira DW. 1994 Cutaneous leishmaniasis in Kenya: *Sergentomyia garnhami* (Diptera Psychodidae), a possible vector of *Leishmania major* in Kitui District: a new focus of the disease. East African Medical Journal, 71, 424–428. 7828493

[29] Ngumbi PM, Lawyer PG, Johnson RN, Kiilu G, Asiago C. 1992 Identification of phlebotomine sandfly bloodmeals from Baringo District, Kenya, by direct enzyme-linked immunosorbent assay (ELISA). Medical and Veterinary Entomology, 6, 385–388. 1463906 10.1111/j.1365-2915.1992.tb00638.x

[30] Nzelu CO, Kato H, Puplampu N, Desewu K, Odoom S, Wilson MD, Sakurai T, Katakura K, Boakye DA. 2014 First detection of *Leishmania tropica* DNA and *Trypanosoma* species in *Sergentomyia* sand flies (Diptera: Psychodidae) from an outbreak area of cutaneous leishmaniasis in Ghana. PLoS Neglected Tropical Diseases, 8, e2630. 24516676 10.1371/journal.pntd.0002630 PMC3916256

[31] Parvizi P, Amirkhani A. 2008 Mitochondrial DNA characterization of *Sergentomyia sintoni* populations and finding mammalian *Leishmania* infections in this sandfly by using ITS-rDNA gene. Iranian Journal of Veterinary Research, 9, 9–18.

[32] Pothirat T, Tantiworawit A, Chaiwarith R, Jariyapan N, Wannasan A, Siriyasatien P, Supparatpinyo K, Bates MD, Kwakye-Nuako G, Bates PA. 2014 First isolation of *Leishmania* from Northern Thailand: case report, identification as *Leishmania martiniquensis* and phylogenetic position within the *Leishmania enriettii* complex. PLoS Neglected Tropical Diseases, 8, e3339. 25474647 10.1371/journal.pntd.0003339 PMC4256172

[33] Ready P. 2013 Biology of Phlebotomine sand flies as vectors of disease agents. Annual Review of Entomology, 58, 227–250. 10.1146/annurev-ento-120811-153557 23317043

[34] Sadlova J, Dvorak V, Seblova V, Warburg A, Votypka J, Volf P. 2013 *Sergentomyia schwetzi* is not a competent vector for *Leishmania donovani* and other *Leishmania* species pathogenic to humans. Parasites & Vectors, 6, 186. 23786805 10.1186/1756-3305-6-186 PMC3751727

[35] Seblova V, Sadlova J, Carpenter S, Volf P. 2012 Development of *Leishmania* parasites in *Culicoides nubeculosus* (Diptera: Ceratopogonidae) and implications for screening vector competence. Journal of Medical Entomology, 49, 967–970. 23025175 10.1603/me12053

[36] Seblova V, Sadlova J, Carpenter S, Volf P. 2014 Speculations on biting midges and other bloodsucking arthropods as alternative vectors of *Leishmania*. Parasites & Vectors, 7, 222. 24884857 10.1186/1756-3305-7-222 PMC4024269

[37] Seblova V, Sadlova J, Vojtkova B, Votypka J, Carpenter S, Bates PA, Volf P. 2015 The biting midge *Culicoides sonorensis* (Diptera: Ceratopogonidae) is capable of developing late stage infections of *Leishmania enriettii*. PLoS Neglected Tropical Diseases, 9, e0004060. 26367424 10.1371/journal.pntd.0004060 PMC4569557

[38] Seccombe AK, Ready PD, Huddleston LM. 1993 A catalogue of Old World phlebotomine sandflies (Diptera: Psychodidae, Phlebotominae). Occasional Papers on Systematic Entomology, 8, 1–57.

[39] Senghor M, Niang A, Depaquit J, Faye M, Ferté H, Elguero E, Gaye O, Alten B, Perktas U, Cassan C, Faye B, Bañuls AL. 2016 Transmission of *Leishmania infantum* in the canine leishmaniasis focus of Mont-Rolland, Senegal: ecological, parasitological and molecular evidence for a possible role of *Sergentomyia* sand flies. PLoS Neglected Tropical Diseases, 10(11), e0004940. 27806051 10.1371/journal.pntd.0004940 PMC5091883

[40] Theodor O. 1948 Classification of the Old World species of the subfamily Phlebotominae (Diptera, Psychodidae). Bulletin of Entomological Research, 39, 85–115. 18865548 10.1017/s0007485300024305

[41] Volf P, Myskova J. 2007 Sand flies and *Leishmania*: specific versus permissive vectors. Trends in Parasitology, 23, 91–92. 17207663 10.1016/j.pt.2006.12.010 PMC2839922

[42] WHO. 2010 Control of the Leishmaniasis: Report of the Who Expert Committee on the Control of Leishmaniases. World Health Organization. WHO Technical Report Series, No. 949 pp. 186 http://www.who.int/neglected_diseases/2010report/NTD_2010report_web.pdf

